# Extracellular matrix remodelling induced by alternating electrical and mechanical stimulations increases the contraction of engineered skeletal muscle tissues

**DOI:** 10.1038/s41598-019-39522-6

**Published:** 2019-02-25

**Authors:** Hyeonyu Kim, Min-Cheol Kim, H. Harry Asada

**Affiliations:** 1 Department of Mechanical Engineering, Massachusetts Institute of Technology, Cambridge, MA 02139 USA; 2 BioSystem and Micromechanics IRG, Singapore-MIT Alliance for Research and Technology, National University of Singapore, Singapore, 117543 Singapore

## Abstract

Engineered skeletal muscles are inferior to natural muscles in terms of contractile force, hampering their potential use in practical applications. One major limitation is that the extracellular matrix (ECM) not only impedes the contraction but also ineffectively transmits the forces generated by myotubes to the load. In the present study, ECM remodelling improves contractile force in a short time, and a coordinated, combined electrical and mechanical stimulation induces the desired ECM remodelling. Notably, the application of single and combined stimulations to the engineered muscles remodels the structure of their ECM networks, which determines the mechanical properties of the ECM. Myotubes in the tissues are connected in parallel and in series to the ECM. The stiffness of the parallel ECM must be low not to impede contraction, while the stiffness of the serial ECM must be high to transmit the forces to the load. Both the experimental results and the mechanistic model suggest that the combined stimulation through coordination reorients the ECM fibres in such a way that the parallel ECM stiffness is reduced, while the serial ECM stiffness is increased. In particular, 3 and 20 minutes of alternating electrical and mechanical stimulations increase the force by 18% and 31%, respectively.

## Introduction

Engineered skeletal muscle tissues (eSMTs) have a variety of applications in the medical and engineering fields. Since skeletal muscle comprises approximately 40% of the human body mass, the development of an *in vitro* skeletal muscle model is important as part of the organs-on-a-chip. Three-dimensional (3D) eSMTs recapitulating the function and structure of natural muscles have been used in preclinical drug discovery^1^ to reduce the costs of drug development and animal tests^2,3^. In addition, eSMTs have been implanted to restore volumetric muscle loss by promoting functional improvement^4–6^. Moreover, eSMTs have been used as actuators for engineering biological machines, such as grasping or walking across a surface using a design strategy and coordinated muscle movements^7–9^. Previously, numerous *in vitro* muscle tissues have been constructed^10–12^, including the fascicle-inspired, 3D eSMTs formed with special sacrificial moulding techniques^13,14^. In these eSMTs, striated *α*-actinin and tetanus have been observed^14^. Nonetheless, their performance in force (less than 10 kPa) is by far inferior to natural skeletal muscles (approximately 250 kPa)^11,15^. Training is a key step in myotube maturation to achieve a higher contractile force of the eSMTs. Previously, electrical or mechanical stimulation has been applied for several days to weeks to obtain more proliferation and differentiation^16–18^. Although those training methods helped to increase the muscle performance, the trained eSMTs still displayed a significantly lower contractile force than natural muscles^19^.

The limited performance of eSMTs is due to several differences between eSMTs and native muscles, such as less differentiated myotubes, a low cell density, less organized sarcomeres, and immature Ca^2+^ handling^20^. In particular, distinct features of the eSMT extracellular matrix (ECM) significantly influence the force production and transmission. The ECM in natural skeletal muscles mainly comprises collagens^21,22^, and each myofibre is circumferentially surrounded by a 3 to 4.5 μm thick ECM layer, called the endomysium^23^. Active force is generated from sarcomeres in myofibres and transmitted to the ECM through costameres^24^. The ends of myofibres inside a fascicle are serially connected to other myofibres, mostly by overlapping with each other^21^ or sometimes through very short collagen-bridged junctions that are less than 10 μm in length, termed intrafascicularly terminating ends^25,26^. Unlike the natural skeletal muscle tissues, eSMTs currently have a higher volume fraction of the ECM gel. eSMTs are usually formed by a mixture of myoblasts and the ECM and then cultured for up to 3 weeks. In the eSMTs, myoblasts fuse to form myotubes and premature myofibres during this period, but they are still thinner and much shorter with less muscle differentiation than natural adult muscles. Thus, the eSMTs have a thicker ECM layer, approximately 5 to 15 μm thickness, between myotubes. Additionally, a multitude of the shorter myotubes is connected serially at the intrafascicularly terminating ends, which are approximately 2 to 100 μm long, where the connecting ECM is much longer than in natural muscles (10 μm). Notably, these differences in the ECM significantly decrease muscle performance^11^. The much thicker ECM of the eSMTs impedes the muscle contraction by increasing stiffness of the ECM network and the nonfunctional fibrotic part^27^. Furthermore, the strength and stiffness of serial connections between the shorter myotubes are critical for the longitudinal transmission of tension. Therefore, the parallel ECM at the side of the myotubes must be soft, but the serially connected ECM at the intrafascicularly terminating ends must be stiff to transmit a higher force.

The ECM network is remodelled as it is exposed to mechanical stress and strain that change fibre orientations and their connectivity. Consequently, while most muscle training is designed to facilitate muscle differentiation, training of eSMTs may cause remodelling of the ECM fibres surrounding the myotubes as well as the ECM connecting myotubes in series. Mechanical stretching generates tensile stress on the whole ECM in the direction of the load. In addition, muscle contraction triggered by an electric potential induces shear stress on the ECM at the side of the myotubes^21,28^ and tensile stress at the intrafascicularly terminating ends^29^. The application of these electrical and mechanical stimulations to the eSMTs may have a significant impact upon the remodelling of the ECM^30,31^.

Inspired by the fact that the regenerating parts of natural muscles are naturally exposed to electric potentials from motor neurons in combination with mechanical stretching from surrounding muscles, we hypothesize that applying a combined electrical and mechanical stimulation in a coordinated manner may produce higher contractile forces in eSMTs than a single type of stimulation. We aim to explain the increase in the contractile force in relation to changes in the ECM network configuration. It is known that mechanical changes in the viscoelastic eSMTs strongly depend on time^32^. Therefore, temporal coordination of electrical and mechanical stimulations is important to produce desired changes in the ECM. In this study, we will investigate how the two types of stimulation should be combined and coordinated in a programmable manner with a certain phase shift and frequency.

**Figure 1:**
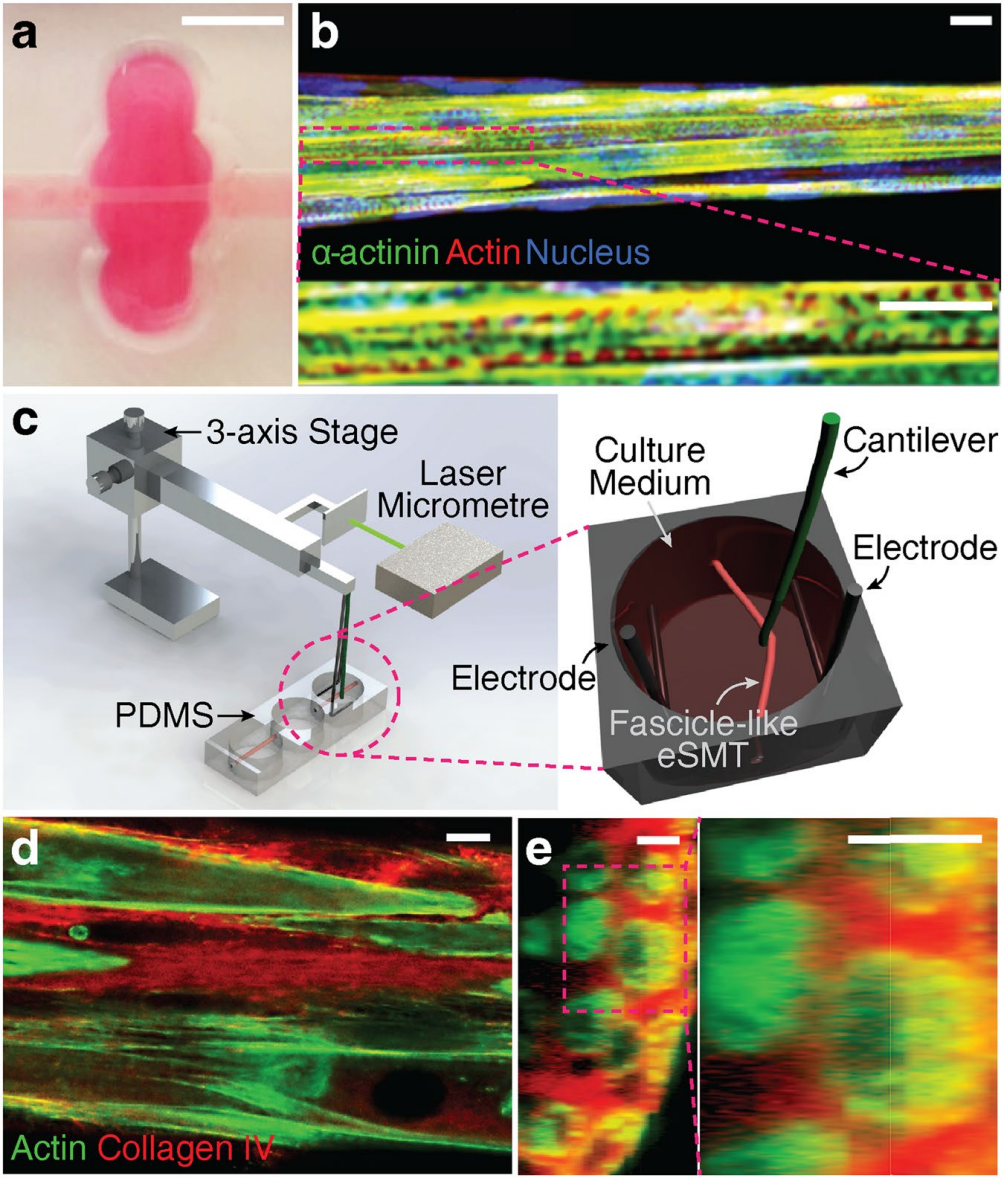
Co-stimulation system and fascicle-inspired engineered skeletal muscle tissue (eSMT). (**a**,**b**) The fascicle-inspired eSMT formed a cylindrical shape with length of 6 mm and diameter of approximately 75 μm (**a**). The tissues were stained using immunofluorescence technique to visualize the striations of *α*-actinin, which is a marker of differentiation and contractility (**b**, reproduced from reference 40 with permission from the Mary Ann Liebert, Inc., New Rochelle, NY). Scale bars represent 5 mm in (**a**) and 10 μm in (**b**). (**c**) Schematic of the experimental setup used to apply coordinated electric and mechanical stimulation to the eSMT. The co-stimulation system consists of electrodes for applying the electric potential, a cantilever wire moved by a servomotor, and a laser micrometre to monitor the displacement of the cantilever. The eSMT is pulled sideways with the cantilever to stretch eSMT to the desired strain. Contractile force was quantified by measuring deformation of the cantilever whose bending stiffness is known. (**d**,**e**) Cross-sectional images of unstimulated eSMTs stained for collagen IV (red) and actin (green) in longitudinal (**d**) and transverse (**e**) directions. Scale bars represent 10 μm.

Previously, several studies have applied static stretching with electrical stimulation to the natural skeletal muscles^33–35^. More studies on combining electrical and mechanical stimulations in a coordinated manner are needed to understand their synergistic effects on muscle training. Recently, *in vitro* systems for combined stimulation were developed for human mesenchymal stem cells^36^, induced pluripotent stem cell-derived human cardiac tissue^37^, rat cardiac cells^38^, and mouse skeletal myoblasts^39^, but no report has described temporal coordination, particularly the alternation of the two types of stimulation for eSMTs, instead of their synchronization. To our knowledge, this study is the first to assess the synergistic coordination of electrical and mechanical stimulation and utilizing it to enhance the contractile force of eSMTs by inducing structural remodelling of the ECM. We used fascicle-inspired 3D eSMTs created with high-density and aligned C2C12s, mouse skeletal muscle myoblasts, and the fibrinogen/Matrigel ECM using a sacrificial moulding technique^40^ (Fig. 1). Several unique features of these eSMTs made them suitable for use in this study. The fascicle-inspired eSMTs only contact a culture medium, without any hard contact except for both ends; this design allows the tissue to have uniform axial stress during an external mechanical stretching and less hindrance to muscle contraction during an electrical stimulation. In addition, a system for measuring the contractile force of the fascicle-inspired eSMTs was developed^40^. We applied the external stimulation to the eSMTs for only 3 minutes to focus on the changes in mechanical properties and reduce possible effects caused by changes at the RNA/protein levels^41^, such as proliferation and differentiation. We built a mechanistic model to better elucidate the effect of ECM remodelling upon the generation and transmission of the eSMT contractile force.

## Results

### Increased contractile forces generated through short-term training with diverse types of stimulation

We developed a testbed that applies combined electrical and mechanical stimulation, which is defined as co-stimulation. Fascicle-inspired eSMTs with pronounced *α*-actinin striation were formed using a 3D sacrificial moulding technique and suspended between two holes (Fig. 1a,b). We modified an existing system^40^ into a new testbed that allowed us to apply mechanical stretching and electric potential, as well as to facilitate contractile force measurements (Fig. 1c and Supplementary Fig. 1). We used a cantilever wire with a known stiffness to measure contractile force and to stretch the eSMT by pulling it in the direction perpendicular to the longitudinal direction of the eSMT. The cantilever was moved by an actuator that was programmed to stretch tissue to the desired strain. We also simultaneously administered an electric potential using the platinum electrodes to achieve combined electrical and mechanical stimulation.

**Figure 2:**
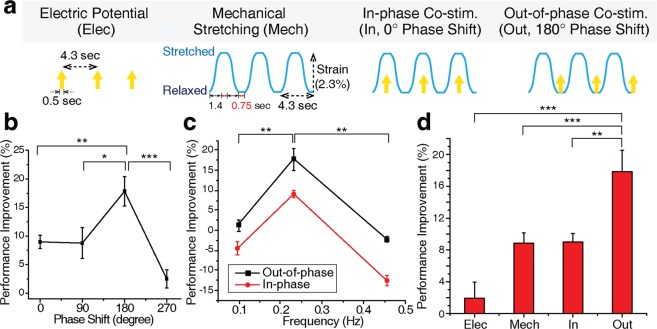
Coordination of the combined electric and mechanical stimulation and synergistic performance enhancement following 3 minutes of stimulation. (**a**) Four patterns of coordination: electrical (Elec) and mechanical (Mech) stimulations alone, and in-phase (In, 0° phase shift) and out-of-phase (Out, 180° phase shift) co-stimulations. (**b**) Improvement in the contractile force induced by the combined stimulation with different phase shifts. SEM, *n* = 10, 5, 10 and 5. (**c**) Improvement in the contractile force induced by stimulations with different frequencies. SEM, *n* = 3, 10, and 3. (**d**) Comparison of the performance improvements in the contractile force induced by the four patterns of stimulation in (**a**), the two single types of stimulation (Elec and Mech) and two co-stimulations (In and Out). SEM, *n* = 9, 11, 10 and 10. Out-of-phase co-stimulation increased the contractile force by 18% in 3 minutes. *P < 0.05, **P < 0.01, and ***P < 0.001.

Both electric potential and mechanical stretching were repeatedly applied to the eSMTs (Fig. 2a). The two periodic stimulations were alternated with phase differences of 0°, 90°, 180°, and 270°. Although an electromechanical coupling of contraction exists^42,43^, the delay time of the coupling is only 30 to 100 ms^42^, which is almost negligible compared to one period of the stimulation used in this study (4.3 sec). The contractile force of the trained eSMTs was normalized by the original contractile force before the training to evaluate the performance improvement. Interestingly, a 180° of phase shift produced the greatest performance improvement and achieved nearly 20% better performance than the 270° phase shift (Fig. 2b), although all other conditions were identical. Furthermore, we applied the co-stimulation at diverse frequencies in similar range reported in previous studies, spanning 0.1–0.5 Hz^44–46^. When we applied the co-stimulations at three frequencies of 0.1, 0.23, and 0.45 Hz, the contractile force was more improved to a greater extent when stimulated at 0.23 Hz (Fig. 2c). In addition, the out-of-phase (180° phase shift) co-stimulation improved the performance more than the in-phase (0° phase shift) co-stimulation, regardless of frequencies (Fig. 2c). Therefore, we chose the out-of-phase co-stimulation at 0.23 Hz as the condition to effectively enhance the contractile force.

Co-stimulation was also compared to individual electrical (Elec) and mechanical (Mech) stimulations (Fig. 2d). The average contractile force of the eSMTs before the stimulation was 4.0 ± 1.7 μN. The performance improvements of single stimulation types were significantly inferior to the out-of-phase co-stimulation (Out), which produced an 18% of improvement after only 3 minutes of training (Fig. 2d). A synergistic improvement was observed in the out-of-phase co-stimulation, compared to the single type of stimulation. The in-phase co-stimulation (In), on the other hand, yielded only an 8.9% improvement, a value that was less than the out-of-phase condition and similar to the mechanical stimulation alone.

### Mechanistic model of a myotube in the eSMT coupled with the surrounding ECM

**Figure 3:**
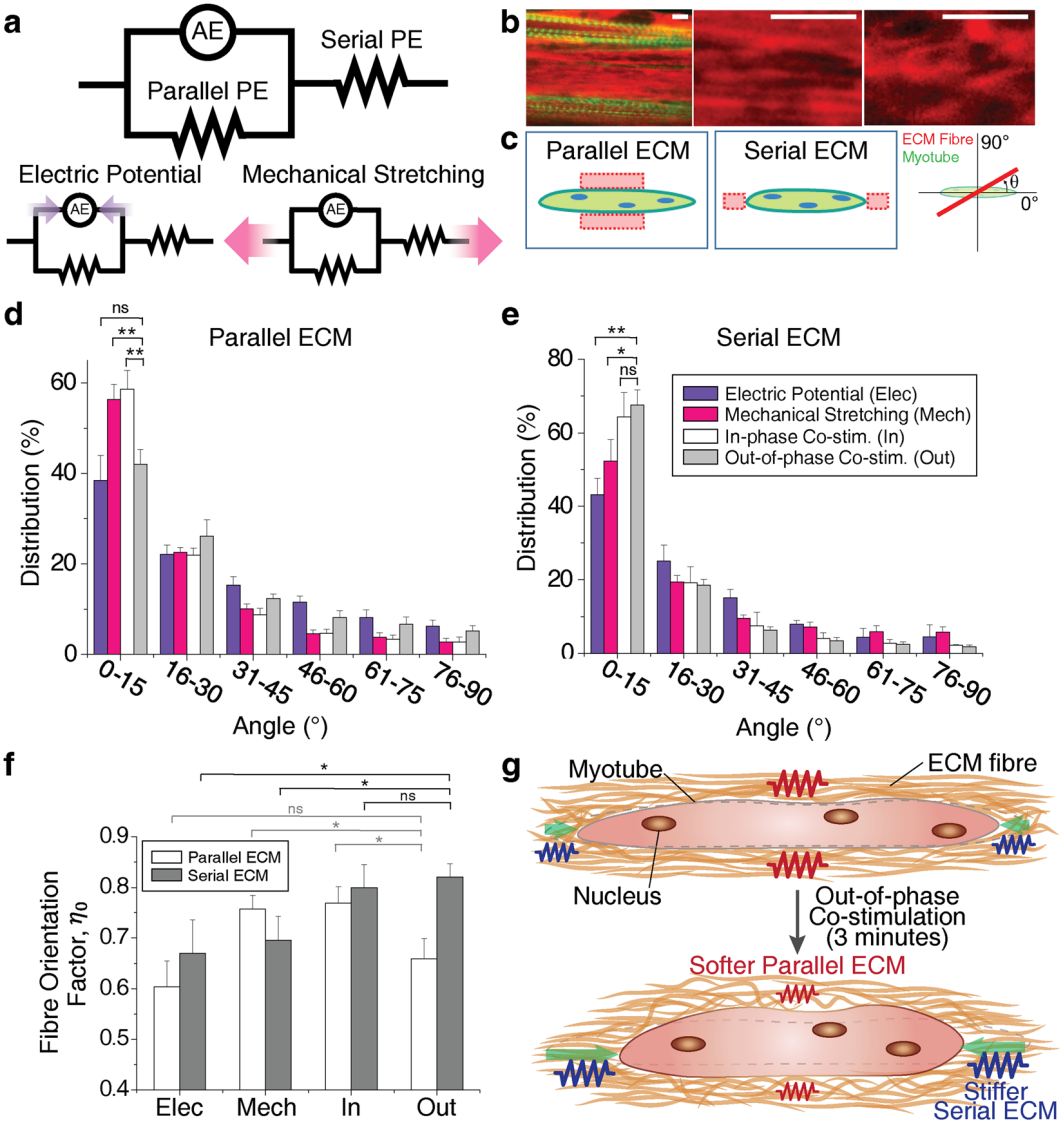
Mechanistic model of eSMT force generation and transmission, and changes in the mechanical property induced by ECM remodelling. (**a**) Mechanistic model of eSMT consisting of an active element (AE), parallel passive element (Parallel PE), and serial passive element (Serial PE). (**b**) Images of collagen IV (red) and actin in the myotubes (green) of the eSMTs to measure the fibre orientation distribution of the ECM network (left). Images of well-aligned collagen fibres (middle) and less-aligned fibres (right). Scale bars represent 5 μm. (**c**) Schematics showing the regions of the parallel and serial ECMs relative to the myotubes, and ECM fibre orientation *θ* measured from the longitudinal direction of myotube. (**d**,**e**) Orientation distribution of collagen IV in the parallel (**d**) and serial (**e**) ECM regions. Most ECM fibres were aligned parallel to the longitudinal direction of the myotubes (0°) by pretension. SEM, *n* = 8, 12, 11, and 10 (parallel), *n* = 3, 7, 3, and 7 (serial). (**f**) Comparison of fibre orientation factors (*η*_*o*_) to predict the elastic modulus of the ECM network (*E*_*L*_). The out-of-phase co-stimulation induced the highest elastic modulus (largest *η*_*o*_) for the Serial PE and a low elastic modulus for the Parallel PE. SEM, n is the same as (**d**,**e)**. *P < 0.05, **P < 0.01, and ns, not significant. (**g**) Schematic depicting the mechanism underlying the changes in performance induced by ECM remodelling following the application of the out-of-phase co-stimulation. The out-of-phase co-stimulation decreases the stiffness of the parallel ECM for less impedance on muscle contraction and increases the stiffness of the serial ECM to increase force transmission to the load.

In an attempt to elucidate the cause of the significant increase in contractile force induced by short-term training, a simple mechanistic model for the myotube in the eSMT was constructed. In the experiments described above, a significant increase in force was obtained in only 3 minutes of the training. This implies that mechanical factors may be responsible for the improvements rather than biochemical factors, which would need more time to cause such significant changes. Myotubes are extensively coupled with the surrounding ECM and, thereby, the net contractile force of the eSMT is highly dependent on the ECM properties. Mechanical factors of the muscle tissues are attributed to active and passive elements (Fig. 3a). Sarcomeres inside the myotube, which generate contractile force, represent the active element (AE). The passive elements (PE) comprise all the components that do not produce a force in the tissue, including the ECM and non-contractile parts of muscle cells. However, a noticeable difference between the actin networks in the eSMTs was not observed following the administration of the different stimulations for 3 minutes (Supplementary Fig. 2), as all of the actin fibres were well-aligned in the longitudinal direction.

As we mentioned above, the ECM of the eSMTs shows distinct features from the ECM of natural skeletal muscles: (a) much thicker ECM layers in parallel to the myotubes, and (b) a much longer serial ECM that connects the two ends of the intrafascicularly terminating myotubes (Fig. 1d,e). These coupled myotubes and ECM properties were represented with a mechanistic model consisting of the parallel and serial passive elements (Parallel PE and Serial PE, respectively) in conjunction with the AE (Fig. 3a). According to this model, as the stiffness of the Parallel PE increases, the displacement induced by the contractile force at the AE decreases. This explains possible impediments of muscle contraction due to the thick ECM, particularly when the ECM is stiffer than myotubes^47^. The mechanistic model also explains that the force generated from the active element cannot be transmitted to the load when the Serial PE is too soft. Therefore, the contractile force generated from the myotubes of the eSMTs is not transmitted well to the end of the tissue if they are connected by a soft serial ECM. In summary, the parallel ECM should be soft, but the serial ECM should be stiff enough to transmit a higher contractile force.

### ECM fibre orientation

Based on the mechanistic model that shows the mechanical effects of the Parallel and Serial PEs on the force generation, we hypothesized that effective structural remodelling of the parallel and serial ECMs is induced by the out-of-phase co-stimulation compared to other training methods (Fig. 2d). We assumed that 3 minutes of the training did not change the composition and amount of the ECM because the time was too short to produce significant quantities of new ECM proteins. We measured the distribution of the ECM fibre orientations of the trained eSMTs to examine the changes in the mechanical property of the ECM network. The endomysium of the natural muscles contains collagen I, III, IV, and VI^48,49^. Collagen IV, which is one of the main components of the muscle ECM, particularly in the ECM surrounding newly formed myotubes during skeletal muscle regeneration^50^, contributes to muscle contraction by attaching to a sarcolemma and connecting the other types of collagen to myotubes^49,51^. In this sense, collagen IV and muscle contraction are significantly influenced by each other through the cell-ECM interaction. In the case of the eSMTs, myoblasts were seeded with a fibrin and the Matrigel matrix to form the tissue. Staining of collagen IV, which is one of the main components of the Matrigel, showed that myotubes were largely surrounded by collagen IV (Fig. 1e). When we imaged fibrin, the training did not produce any noticeable change in fibrin, which consists of a highly aggregated network (Supplementary Fig. 3). Therefore, we observed collagen IV near the myotubes to study the effects of the structural remodelling of the ECM induced by the stimulation on the contractile performance.

After we trained the eSMTs with the four different methods shown in Fig. 2d, the collagen IV network of the eSMTs was imaged (Fig. 3b) to measure the fibre orientation distributions at the parallel and serial ECM regions (Fig. 3c). The orientations of the fibres were mostly aligned in the longitudinal direction of the myotubes (0°) due to the pretension generated during the formation of the fascicle-inspired eSMTs (Fig. 3d,e). From the orientation distributions, we compared the elastic modulus of the ECM network (*E*_*L*_) in the longitudinal direction to the myotubes using the rule of mixtures: *E*_*L*_ = *η*_*o*_*E*_*f*_*V*_*f*_ + *E*_*m*_*V*_*m*_, where *η*_*o*_ is fibre orientation factor, *E*_*f*_ is the elastic modulus of the single ECM fibre, *E*_*m*_ is the elastic modulus of medium, *V*_*f*_ is the volume fraction of the ECM fibre, and *V*_*m*_ is the volume fraction of medium^52^. We set *θ* to the angle of the ECM fibre with respect to the longitudinal direction of the myotubes (Fig. 3c) and *f*(*θ*) to the proportion of fibre content in the angle *θ*. The fibre orientation factor was calculated as ${\eta }_{o}=\int \,f(\theta ){\cos }^{4}\theta d\theta$. The value of this equation becomes 1 when all the fibres in the network are aligned in the loading direction (0°) and 0.375 for randomly oriented fibres. We assumed that 3 minutes of the training did not influence *E*_*f*_ or *V*_*f*_, and thus the dominant change caused by the training was the fibre orientation. Since *E*_*m*_ for the liquid medium in the network is almost zero, the *E*_*m*_*V*_*m*_ term is negligible. Therefore, we compared *E*_*L*_ of the parallel and serial ECM regions in the trained eSMTs by calculating the fibre orientation factor *η*_*o*_ (Fig. 3f).

### Changes in ECM fibre orientation of the eSMTs upon stimulation

Differences in the distributions of fibre orientations shown in Fig. 3d,e indicate that each type of stimulation exerted a different effect on the structural remodelling of the ECM. As summarized in Fig. 3f, the out-of-phase co-stimulation generated the highest fibre orientation factor for the collagen IV network of the serial ECM and the low orientation factor for the parallel ECM. Therefore, the out-of-phase co-stimulation induced the greatest stiffness in the serial ECM and a low stiffness for the parallel ECM, both of which are desired for effectively generating and transmitting a contractile force (Fig. 3g). According to Fig. 3e, the electrical stimulation alone did not adequately align the fibres of the serial ECM. On the other hand, the mechanical stimulation alone resulted in more aligned fibres of the serial ECM, but the alignment was less than that induced by the co-stimulations. In addition, the mechanical stimulation increased the alignment in the parallel ECM (Fig. 3d), which is not desirable. The in-phase co-stimulation also aligned more fibres of the parallel ECM than other stimulations. Therefore, as shown in Fig. 3, the out-of-phase co-stimulation most effectively increased performance by promoting the desired structural remodelling of the ECM, particularly by differently altering the ECM depending on its location relative to the myotube. Furthermore, these ECM remodelling improved both the contractile force and stability of the contractile force by switching from the unfused tetanus to fused tetanus (Supplementary Fig. 4).

### Effect of the short-term stimulation on the active element

**Figure 4:**
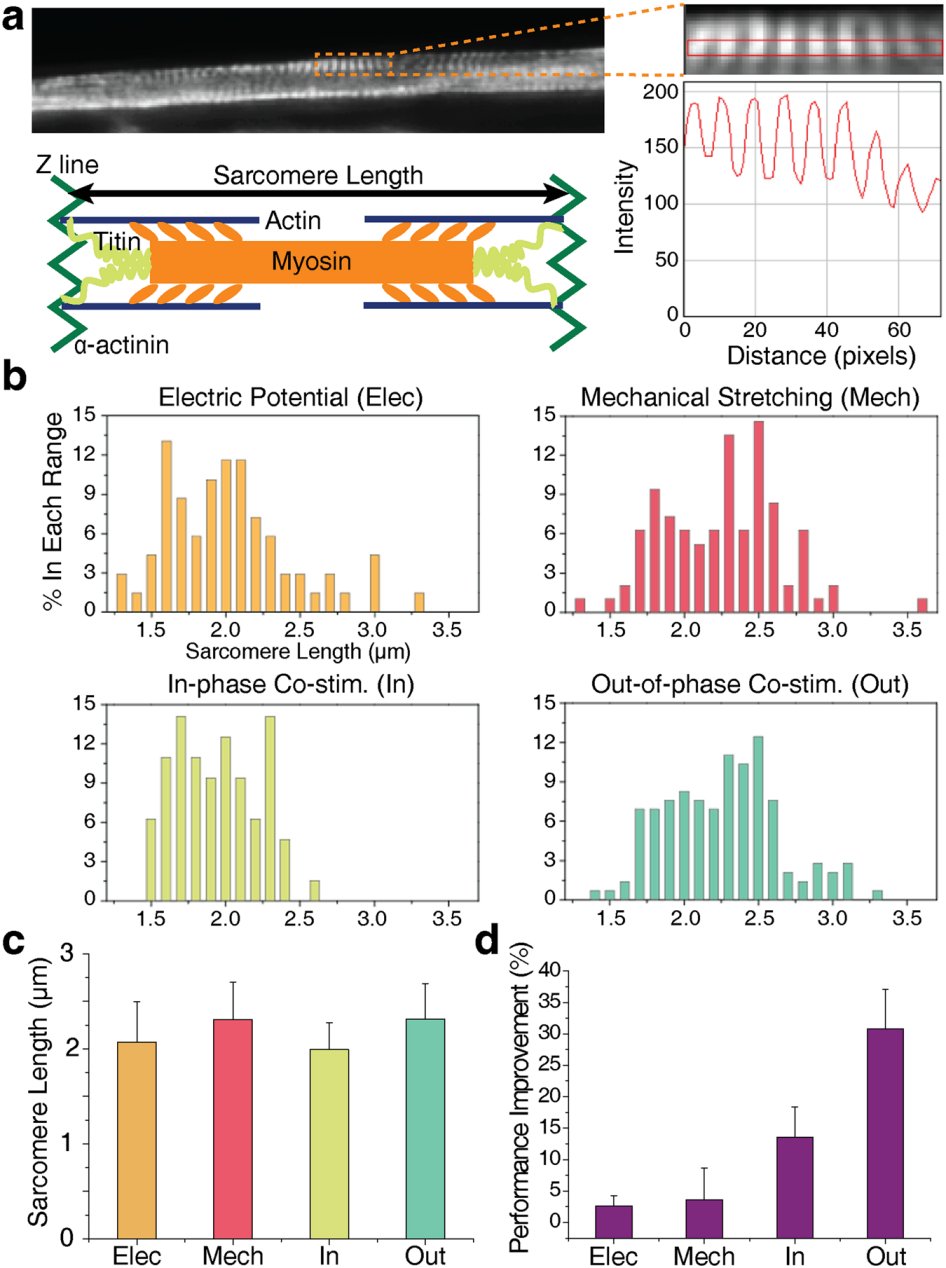
Investigation of changes in the active element and long-term (20 minutes) effects of the stimulation. (**a**) Measurement of sarcomere length based on the intensity of *α*-actinin immunostaining images. The schematic shows the sarcomere structure and length. (**b**) Percentage (%) distributions of sarcomere length in each range after applying the electric potential (Elec), mechanical stretching (Mech), in-phase co-stimulation (In), and out-of-phase co-stimulation (Out) for 3 minutes. (**c**) Average sarcomere length after applying the four different types of stimulations: Elec, Mech, In, and Out. SD, *n* = 69, 96, 64, and 145. (**d**) Longer-term performance enhancement of eSMTs following the application of the four stimulations for 20 minutes, SEM, *n* = 3 for all. Out-of-phase co-stimulation enhanced the contractile force by 31% in 20 minutes.

In addition to the changes in the passive elements, the active element might also contribute to the improvements in the force generated by the eSMTs (Fig. 3a). A sarcomere has an optimal range of lengths to produce a high active tension with optimal actin-myosin overlap, that is, 2 to 2.4 μm^53^. Therefore, we measured the sarcomere length in the trained tissues (Fig. 4a) to investigate the changes in the active element. Since the sarcomere length is the distance between adjacent Z-lines, we stained *α*-actinin of the trained eSMTs, a key component of the Z-line (Fig. 4a). The average sarcomere lengths after applying the mechanical stimulation and out-of-phase co-stimulation were 2.3 μm, and the lengths after applying the electrical stimulation and in-phase co-stimulation were 2.1 and 2.0 μm, respectively (Fig. 4b,c). Because all the lengths were ranged from 2 to 2.4 μm, the differences in the force generation due to the active element following the four different types of stimulations (Fig. 2d) were negligible. Consequently, the performance enhancement induced by the short-term stimulation resulted mostly from changes in the passive elements, specifically through the ECM remodelling.

The four types of stimulations were administered for 20 minutes to examine the effects of longer stimulations. We obtained a 31% performance improvement for the out-of-phase co-stimulation (Fig. 4d), a value that was 1.7 times higher than the improvement induced by the 3-minute stimulation. Applying the co-stimulation for 20 minutes may induce more remarkable ECM remodelling than the 3-minute stimulation. This structural change in the ECM, which alters its mechanical properties, may affect muscle differentiation induced by mechanotransduction on much longer time scale than 20 minutes, as substrate stiffness has been shown to play an important role in muscle differentiation^54^.

## Discussion

Fascicle-inspired eSMTs improved the generation of contractile forces by 18% in 3 minutes and 31% in 20 minutes following alternating the two types of stimulation, electric potential and mechanical stretching. In contrast, the single type of stimulations produced significantly lower improvements: only 2% for the electric potential alone and 8.8% for mechanical stretching alone after 3 minutes of the stimulation. The sum of these two numbers is only 10.8%, which is approximately half of the increase in performance induced by the out-of-phase co-stimulation. Thus, alternating the two types of stimulation produces a synergistic effect. In addition, applying the in-phase and out-of-phase co-stimulations resulted in differences in ECM remodelling at the eSMTs, although they were subjected to identical electrical and mechanical stimulations.

Mechanical stretching applies a tensile stress to the eSMT, while electric potential causes contraction, which induces shear and compressive stresses. In the co-stimulation, where the two single stimulations are combined, temporal coordination of the various stresses is important to induce the appropriate ECM remodelling. In particular, in the parallel ECM, which should be less aligned in the myotube direction not to hinder the muscle contraction, the in-phase co-stimulation develops both the shear stress and tensile stress simultaneously, which can create the highest shear stress, resulting in the highest parallel ECM alignment (the highest percentage of fibre distribution between 0 and 15 degrees). On the other hand, in the out-of-phase condition, this alignment is avoided by alternately administrating the two single stimulations. This difference leads to more ECM alignment of the parallel ECM in the myotube direction by the in-phase co-stimulation than the out-of-phase co-stimulation (Fig. 3d). In addition, the two co-stimulations further align the serial ECM to a greater extent (Fig. 3e) than the single stimulations (electric potential and mechanical stretching). This finding might be explained by the bi-polar, push-pull effects of alternating these two single stimulations on the ECM network, which can yield a grater ECM alignment than mono-polar effects induced by the single stimulations. Moreover, the ECM is a viscoelastic material with the characteristics of strain stiffening and stress relaxation. These properties will behave differently during the application of the two co-stimulations, which have different temporal coordination of the various stresses and result in the different ECM remodelling.

Although the out-of-phase co-stimulation produces more desirable ECM remodelling than the other stimulations, the difference between eSMTs and the natural skeletal muscles is still significant. The endomysium of natural muscles comprises a quasi-random network of wavy ECM fibres^21^. The ECM of the eSMTs was mostly aligned in the longitudinal direction to myotubes (Fig. 3d,e), because of a pretension between the fixed ends of the eSMTs generated by cell-mediated gel compaction. This ECM orientation was similar to the one of highly extended natural muscles^31^. In addition, the serially connected ECM at the intrafascicularly terminating myotubes should be stiff in the eSMTs for force transmission, but this is not required for the case of the natural muscles with myotubes of a sufficient length, which allows to have myotube overlapping. In some regions, the serial ECM was also the parallel ECM for other myotubes. In this case, the ECM might be neither too stiff nor too soft to generate the force from the tissues. Moreover, the incorporation of fibroblasts, the major source of the endomysium, and the endomysium-embedded blood vessels into the eSMT will help to more closely mimic the mechanical and chemical environment of the endomysium in native muscles. We also used the myoblast cell line (C2C12) to prepare the eSMTs that have been used in this study. If primary myoblasts were used, the eSMTs would have greater heterogeneity owing to donors variability, a lower myotube fusion rate because of a hindrance by non-myoblasts cells in cell population, and the ECM with distinct thickness and composition due to the influence of the non-myoblast cells. On the other hand, performing experiments with primary cells would allow us to study donor characteristics and mimic the tissue complexity caused by various types of cells. Therefore, future studies using primary cells would contribute to creating a personalized drug testing platform and understanding the diversity of native muscles.

An improvement in performance was obtained in only 3 minutes, but further investigations are needed to identify other possible mechanisms in addition to ECM remodelling. During such a limited time of 3 minutes, many mechanical and biological factors could be altered by the external stimulations, such as the production of reactive oxygen species (ROS)^55^, opening of the stretch-activated channels (SACs), and cell fluidization^56^. However, changes in the ROS levels and opening of the SAC do not substantially affect the muscle contractile force in such a short time^57,58^. Cell fluidization is characterized by an immediate reduction in F-actin levels right after stretching of the cells, but the F-actin levels are recovered completely within 5 minutes^56^. On the other hand, our results show that the performance improvement of the trained eSMTs maintained for 3 hours (−0.269 ± 4.25% change, *n* = 4). Applying mechanical or electrical stimulation can also activate some translation/transcription-related mechanisms, such as PI3K/Akt/TOR signalling^59^ and nitric oxide-activated proliferation^60^. However, transcription and translation speeds^61^ are too slow to facilitate performance improvement in the stimulation time of 3 minutes. Therefore, we conjecture that the increase in contractile force of the eSMTs observed in a short time is likely due to the ECM remodelling rather than these biological changes. Even so, further investigation is required to investigate other possible mechanisms that are potentially activated by the short-term stimulations.

In this paper, we developed a method for increasing the contractile force of the eSMTs through short-term stimulation. Alternating mechanical and electrical stimulations resulted in approximately a 20% higher contractile force in 3 minutes when the two stimulations are alternated with a 180° phase shift at a frequency of 0.23 Hz. In an attempt to explain the mechanism underlying the synergistic improvements induced by the co-stimulation, we generated a mechanistic model based on the morphology of the myotubes and the surrounding ECM in the eSMTs. We examined changes in both passive and active elements involved in the mechanistic model by measuring the orientation distribution of ECM fibres and the length of sarcomeres after applying the stimulation. This model and the experimental results showed how the out-of-phase co-stimulation mechanically enhanced the contractile force by inducing desirable ECM remodelling and cell-ECM interactions for force generation. We obtained two insights from these results: 1) coordinating and harmonizing the two stimulations that produce bi-polar effects is important to produce a higher contractile force of engineered muscles; and 2) the contractile force of engineered muscles is significantly improved not only by biological changes, such as differentiation and proliferation but also by mechanical changes. This work will contribute to overcoming the performance limitation of the engineered muscles and meeting the requirements for eSMTs to serve as a drug testing platform and actuators of micro bio-bots.

## Methods

### Cell culture

The C2C12 mouse myoblast cell line (ATCC, Manassas, VA, USA) was used in this study. Myoblasts were maintained in the growth medium (GM) comprising Dulbecco’s modified eagle medium (DMEM, Sigma-Aldrich, St. Louis, MO, USA) supplemented with 10% fetal bovine serum (FBS, Sigma-Aldrich, St. Louis, MO, USA), 1% penicillin-streptomycin (PS, Sigma-Aldrich, St. Louis, MO, USA), and 0.2% Normocin (InvivoGen, San Diego, CA, USA). Cells were passaged when they reached 70% confluence to ensure that the cells remained unfused. The growth media was changed daily after we seeded the cells in the fabricated chip to form the fascicle-inspired eSMTs.

### Fascicle-inspired eSMTs

The 3D fascicle-inspired muscle constructs used in the current study were formed using the sacrificial moulding technique^14^. Myoblasts were mixed with fibrinogen (Sigma-Aldrich, St. Louis, MO, USA) and growth factor-reduced Matrigel (Corning, NY, USA) and were seeded in the fabricated chip to form the fascicle-inspired eSMTs (see the Supplementary Methods). Two days after seeding, the cells were cultured in the GM with 1 mg/ml aminocaproic acid (AA, Sigma-Aldrich, St. Louis, MO, USA). AA was added to mitigate ECM degradation. Beginning on day 3, we changed the medium from GM to differentiation medium (DM) supplemented with 1 mg/ml AA to induce muscle cell differentiation *in vitro*. The DM was the same as GM except for the 4% horse serum instead of the fetal bovine serum (FBS). The medium was changed daily for 10 days.

### Measurement of the contractile forces of skeletal muscles

Using a single apparatus, we not only applied the co-stimulation, but also quantified the concentric contractile force (see the Supplementary Methods). First, we stretched muscles (2.3% strain) using the tip of the cantilever (Enameled Copper 33 AWG, Remington Industries, Johnsburg, IL, USA) to allow muscles to shorten during the concentric contraction. Then, the tension of the stretched muscle and the restoring force of the cantilever were balanced (Supplementary Fig. 1). When we also applied the electric potential, the tip was displaced because of the additional muscle contractile force towards the original position of the muscle during shortening (Supplementary Fig. 1).

### Co-stimulation system

A programmable co-stimulation system was constructed by integrating an electric device required to generate an electric potential and a motor-driven stage (Thorlabs, Auburn, CA, USA) for stretching 3D eSMTs, both of which were controlled by a computer (Fig. 1c). The electric potential was administered with two platinum wires placed in the medium beside the muscle tissue using a 3D printed wire holder (Dimension 1200es, Stratasys, Eden Prairie, MN, USA). An electrical field of 2.5 V/mm was applied with bipolar pulses of 1 ms each for 0.5 seconds per time for training. The two electrodes were placed parallel to the longitudinal direction of the eSMT, and the electric field was generated between the electrodes perpendicular to their direction. Mechanical stretching was produced through the cantilever wire (Enameled Copper 33 AWG, Remington Industries, Johnsburg, IL, USA) by pulling the muscle sideways (Supplementary Fig. 1 and Supplementary Movie).

### Immunostaining

Collagen IV was immunostained with primary antibodies against collagen IV (Sigma-Aldrich, St. Louis, MO, USA) and *α*-actinin (Thermo Fisher Scientific, Waltham, MA, USA) after fixing the trained eSMTs with 4% paraformaldehyde (Santa Cruz Biotechnology, Dallas, TX, USA), and permeabilizing them with 0.2% Triton-X (Thermo Fisher Scientific, Waltham, MA, USA). Trained eSMTs were incubated with 1% bovine serum albumin (Sigma-Aldrich, St. Louis, MO, USA) to block the nonspecific binding of antibodies. Hoechst (Thermo Fisher Scientific, Waltham, MA, USA) and rhodamine phalloidin (Thermo Fisher Scientific, Waltham, MA, USA) were applied together for 30 minutes to visualize nucleus and actin, respectively.

### Measurement of the orientation distribution of ECM fibres and sarcomere length

We used the ImageJ plugin OrientationJ^62^ and DDecon (The Scripps Research Institute, La Jolla, CA) to measure the orientation distributions of the collagen IV fibres and sarcomere length in the immunostaining images, respectively.

### Statistical analyses

Statistical analyses were conducted using Student’s t test. Data were considered statistically significant if the P-value was 0.05 (*), 0.01 (**), or 0.001 (***).

## Supplementary information

Supplementary Information

Supplementary Movie
