# Supplementary material for: Extracellular matrix remodelling induced by alternating electrical and mechanical stimulations increases the contraction of engineered skeletal muscle tissues
Source: Sci Rep. 2019 Feb 25;9:2732. doi: 10.1038/s41598-019-39522-6 (PMC6389954; doi:10.1038/s41598-019-39522-6)
Supplement: Supplementary file 1 — Supplementary Information [file 41598_2019_39522_MOESM1_ESM.pdf]

Supplementary Information

Extracellular matrix remodelling induced by alternating electrical and mechanical stimulations increases the contraction of engineered skeletal muscle tissues

Authors: Hyeonyu Kim[1,*], Min-Cheol Kim[1], H. Harry Asada[1,2]

[1]Department of Mechanical Engineering, Massachusetts Institute of Technology, Cambridge, MA 02139, USA.
[2]BioSystem and Micromechanics IRG, Singapore-MIT Alliance for Research and Technology, National University of Singapore, 117543, Singapore.
*Correspondence and requests for materials should be addressed to H.K. (email: hyeonyu@mit.edu).

Supplementary Figures

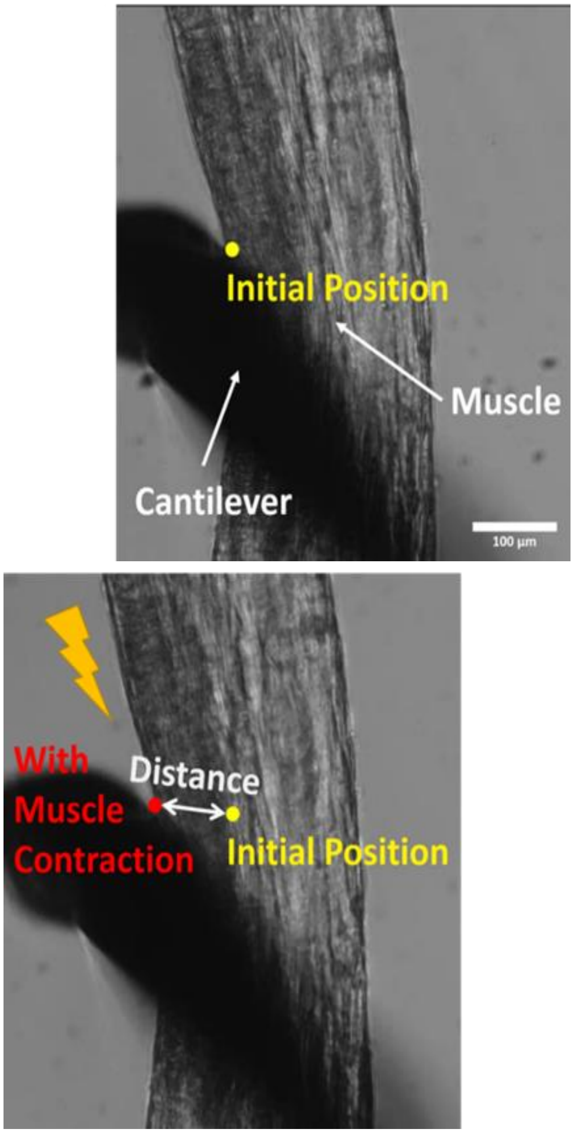

Supplementary Figure 1. Measurement of the muscle contractile force. These images are an enlargement of the contact point between the eSMT and the tip of cantilever in Fig. 1c during muscle contraction. The point of the force balance between the elastic force of the cantilever wire and the forces from the eSMT is shifted by inducing muscle contraction, and the moving distance of the cantilever is proportional to the contractile force. Yellow dot is original force equilibrium position, and the yellow dot is shifted to the red one by the contractile force.

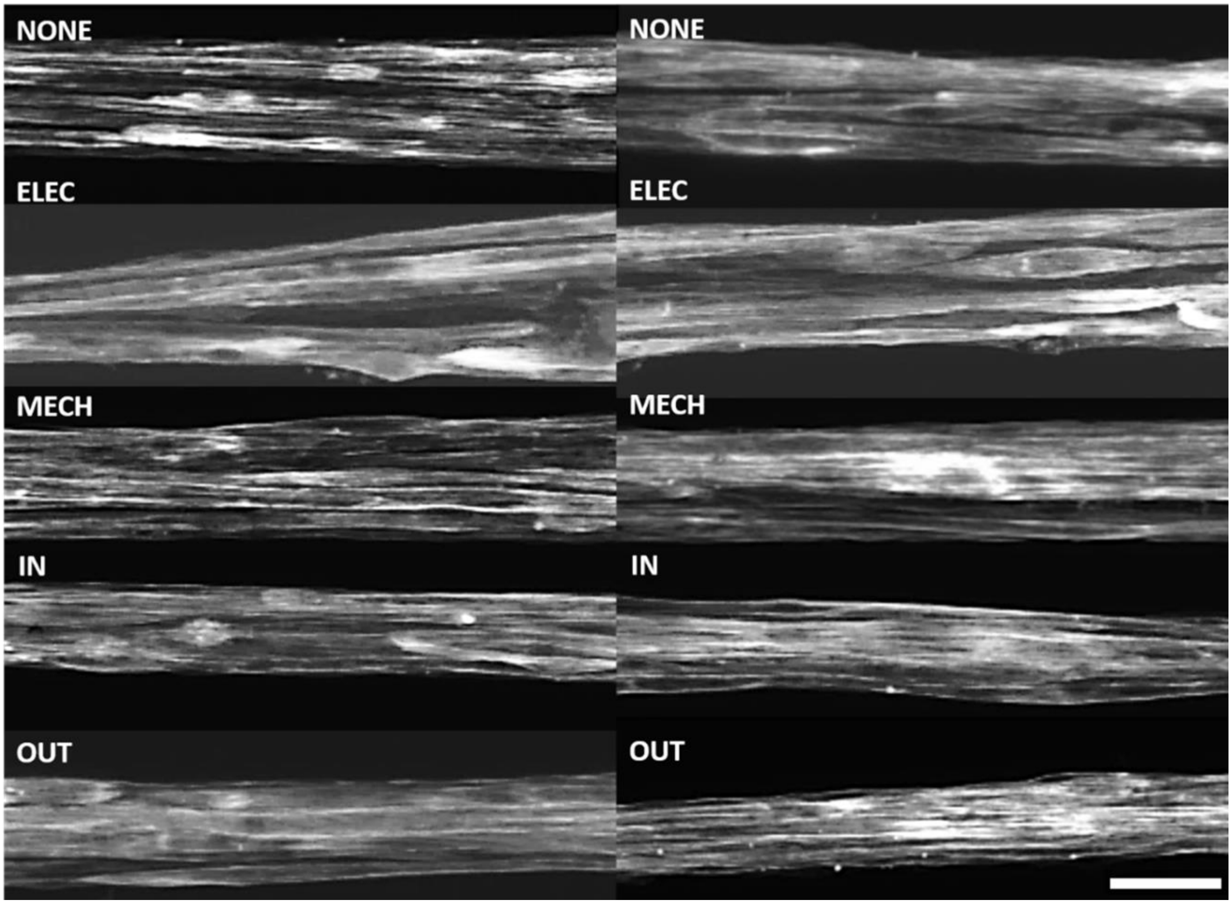

Supplementary Figure 2. Immunostaining images of actin in the eSMTs following the application of the electric potential (ELEC), mechanical stretching (MECH), in-phase co-stimulation (IN), and out-of-phase co-stimulation (OUT) for 3 minutes. There was no notable difference in the actin networks between the 3-minute stimulation conditions. Scale bar represents 100 μm.

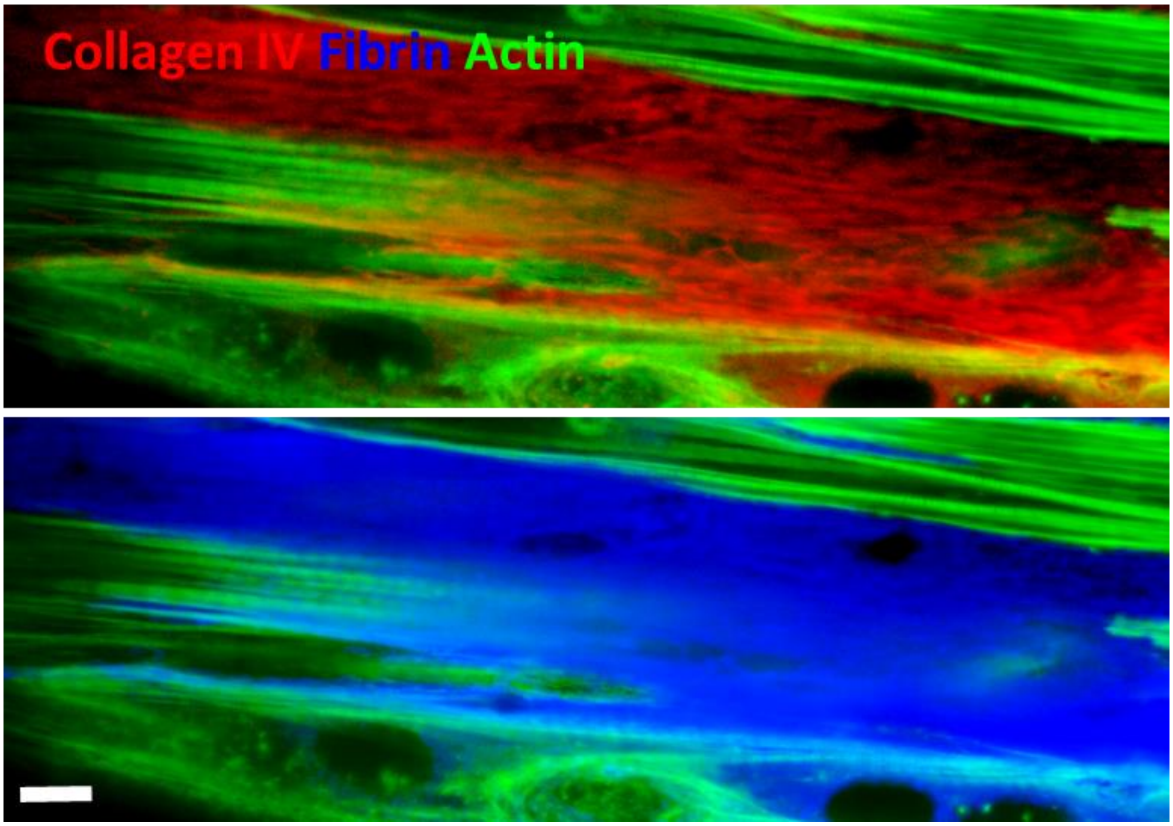

Supplementary Figure 3. Immunostaining images of collagen IV (red), fibrin (blue), and actin (green) in the unstimulated eSMT. Fibrin was much more aggregated than collagen IV. Scale bar represents 10 μm.

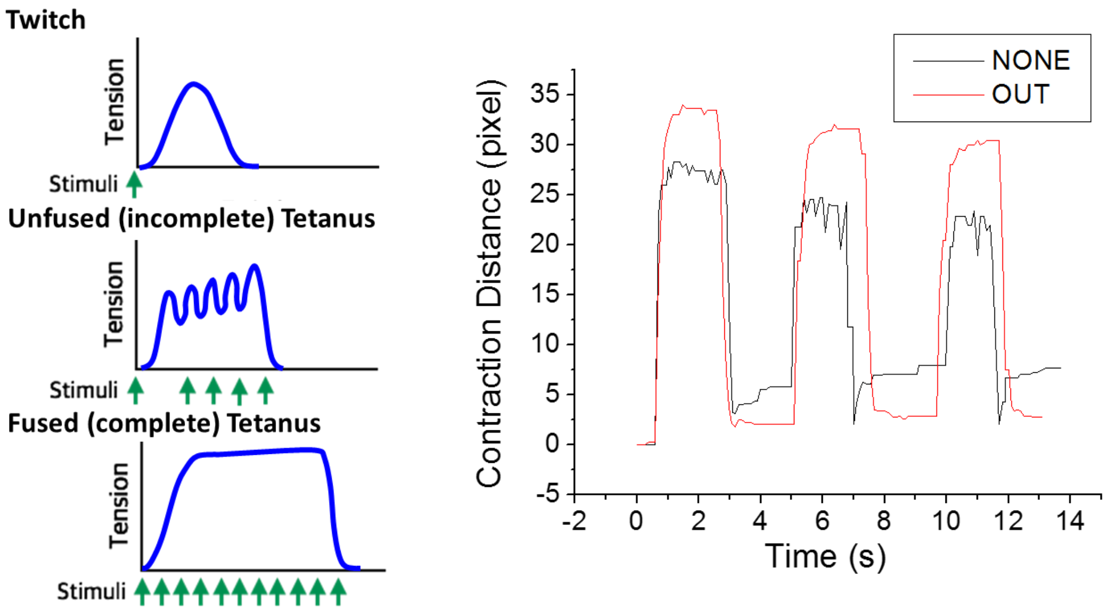

Supplementary Figure 4. An unfused tetanus of the untrained muscles became a fused tetanus by applying 3 minutes of the out-of-phase co-stimulation. (Left) The concept of the twitch, unfused tetanus and fused tetanus. In the general case of the native muscles, they show twitch, unfused or fused tetanus according to the frequency of the applied electrical stimulation. (Right) Although the stimulation frequency (2.5 V/mm, 1 ms, 60 Hz) was same, the unfused tetanus changed to the fused tetanus after applying the out-of-phase co-stimulation to the eSMT for 3 minutes.

Supplementary Movie. Animation to explain the co-stimulation system, which can give the combined electrical and mechanical stimulation, and also can measure contractile force.

Supplementary Methods

Fascicle-like engineered skeletal muscle tissue

In this paper, we used the fascicle-like eSMT. The eSMT made using this new technique is a 6 mm long and about 0.1 mm thick well-aligned tissue in three-dimensional (3D) environment. This special tissue is only anchored at both ends, and there is no hard contact in the middle of the tissue. Therefore, this enables improved diffusion of medium into the eSMT, and the whole tissue has the uniform tensile stress by cell-mediated gel compaction. There were multiple steps to make the fascicle-like eSMT. Steel pin with nominal diameter 508 μm were inserted into the mold that was printed using 3-D printer (Dimension 1200es, Stratasys, Eden Prairie, MN, USA). After poured Polydimethylsiloxane (PDMS) was solidified in the mold, removing the steel pins made the tubular holes in the PDMS. 6 mm holes were formed using a punch. The size of the holes decides the length of the muscle tissue and the holes were used as a reservoir for medium. The PDMS chips were cut into small pieces with 3 holes each, and bonded with 0.5 mm thin PDMS film. Before putting the cells into the fabricated chips, the chips were autoclaved in order to prevent contamination. One of the key techniques for the fascicle-like device was the sacrificial molding to make tubular shape of the eSMT. The material for the sacrificial mold should be kept solid at blow a certain temperature and form desired shape. However, it should be melted at high temperature to remove the sacrificial mold. Thus, we used 5% gelatin solution melted at 37°C to growth medium and solidified at room temperature The gelatin solution also contained thrombin to use fibrin as the extracellular matrix (ECM), and 0.05 M NaOH solution for pH adjustment. This gelatin solution was poured into the PDMS chips with a pin, and the chips were in the refrigerator for 30 minutes to accelerate gelatin solidification. The trypsinized C2C12 mouse myoblasts were centrifuged, and the cell pellets were mixed with 80% fibrinogen (Sigma-Aldrich, St. Louis, MO, USA), 20% growth factor reduced matrigel (Corning, NY, USA), and cold growth medium (DMEM, Sigma-Aldrich, St. Louis, MO, USA). After removing the pins from the gelatin-solidified chips, the cell and gel mixture was injected in the holes of the gelatin sacrificial mold. In the 37°C incubator, gelatin-thrombin solution was melted and formed the fibrin matrix. Remaining gelatin was diluted out through medium change every day. For two days after seeding, the cells were cultured in GM with 1 mg/ml aminocaproic acid (AA, Sigma-Aldrich, St. Louis, MO, USA). AA was added to mitigate ECM degradation. After that, we changed the media into differentiation medium (DM) with 1 mg/ml AA to induce muscle cell development in vitro. DM was the same as GM except for 4% horse serum instead of the fetal bovine serum (FBS). The medium was changed every day for totally 10 days.

Contractile force measurement of the engineered skeletal muscle tissue

Using the single apparatus, we not only applied the co-stimulation to the eSMT, but also measured its contractile force. Unlike the isometric force measurements in previous researches inhibits shortening of muscle tissues, we measured the concentric contractile force with shortening, which is a more natural way to contract. To allow muscle to shorten during contraction, first we stretched muscle (2.3% strain) using the tip of the cantilever wire (Enameled Copper 33 AWG, Remington Industries, Johnsburg, IL, USA). Then, a tension of the stretched eSMT and a restoring force of elastic wire were balanced

(Supplementary Fig. 1). When we apply the electric potential (2.5 V/mm, bipolar pulses of 1 ms each, for 3 seconds) additionally, the wire tip is displaced because of the additional contractile force towards muscle’s original position during the shortening (Supplementary Fig. 1). We use the same cantilever wire for all the results here, thus the stiffness of the tip kept the same value. We track the displacement of the wire tip using the tracker program (comPADRE). In Supplementary Fig. 1, yellow dot is the original force equilibrium position, and the dot is shifted to the red dot by the contractile force. The distance between the two dots is proportional to muscle contractile force.
